# Multi-Omics of Single Cells: Strategies and Applications

**DOI:** 10.1016/j.tibtech.2016.04.004

**Published:** 2016-08

**Authors:** Christoph Bock, Matthias Farlik, Nathan C. Sheffield

**Affiliations:** 1 CeMM Research Center for Molecular Medicine of the Austrian Academy of Sciences, Vienna, Austria; 2 Department of Laboratory Medicine, Medical University of Vienna, Vienna, Austria; 3 Max Planck Institute for Informatics, Saarbrücken, Germany

**Keywords:** single-cell analysis, combined genome/epigenome/transcriptome/proteome/metabolome mapping, bioinformatic methods, cell state profiling, single-cell systems biology, molecular medicine

## Abstract

Most genome-wide assays provide averages across large numbers of cells, but recent technological advances promise to overcome this limitation. Pioneering single-cell assays are now available for genome, epigenome, transcriptome, proteome, and metabolome profiling. Here, we describe how these different dimensions can be combined into multi-omics assays that provide comprehensive profiles of the same cell.

Sequencing-based assays yield genome-wide data, but at the cost of averaging across large cell populations and ignoring biologically relevant variability at the level of individual cells. By contrast, imaging-based methods, such as fluorescence microscopy and flow cytometry, provide single-cell resolution, but only for a handful of preselected markers.

Rapid technological progress is closing this gap, giving rise to powerful assays for genome-wide profiling in single cells. Single-cell sequencing of genomes [1] and transcriptomes [2] is already well established and broadly useful, and the first methods for mapping single-cell epigenomes [3], proteomes [4], and metabolomes [5] are now becoming available. Combining several of these technologies into integrated multi-omic assays of the same single cells will yield unprecedented insights in fundamental biology and biomedicine.

## Strategies

**Figure 1:**
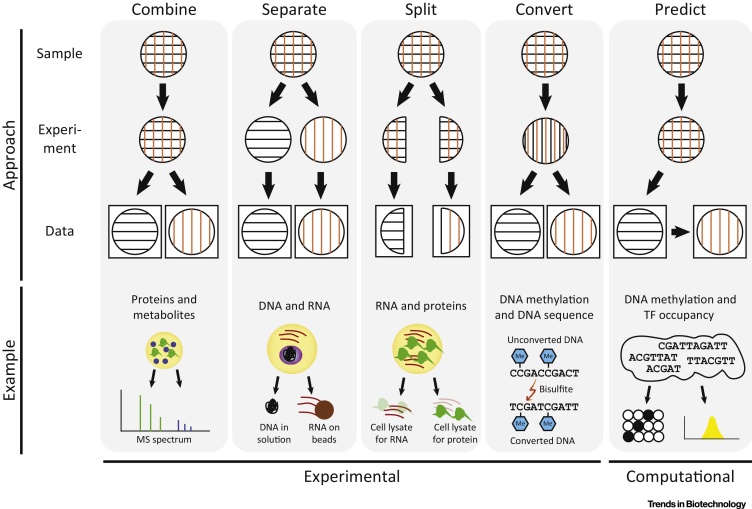
Strategies for Multi-Omics Profiling of Single Cells. Conceptual diagram (top) and examples (bottom) showing five complementary strategies for measuring two different omics dimensions (represented by horizontal and vertical lines) in the same cell. The ‘Combine’ approach measures both dimensions in the same experiment (example: protein and metabolite profiles measured by mass spectrometry). The ‘Separate’ approach enriches two types of biomolecule in different fractions and analyzes them in separate experiments (example: DNA and RNA separated with beads). The ‘Split’ approach uses a fraction of the total cell lysate for each experiment (example: RNA and protein analyzed based on different fractions). The ‘Convert’ approach transforms one omics dimension to another and then analyzes the latter (example: DNA methylation and DNA sequence). The computational ‘Predict’ approach measures one omics dimension directly and bioinformatically infers the second based on the data for the first (example: DNA methylation and transcription factor occupancy). Collectively, these approaches provide building blocks that can be adapted and combined to design protocols for integrated analysis of the genome, epigenome, transcriptome, proteome, and/or metabolome of single cells.

In contrast to fluorescence-based live cell imaging, omics methods, such as next-generation sequencing and mass spectrometry, destroy a cell to analyze it. The first generation of single-cell assays selectively measured a single type of biomolecule (such as DNA, RNA, chromatin, proteins, or metabolites) while discarding all other material. However, there is now proof-of-concept that several omics dimensions can be analyzed in parallel in the same cell; for example, genome/transcriptome or gene/protein levels. We have identified five basic strategies for the multi-omics profiling of single cells (Figure 1).

### Combine

Assays that operate on the same or similar biomolecules may be combined into a single protocol. For example, sequencing methods based on nanopores and single molecule, real-time (SMRT) technology result in kinetic profiles that not only reflect the DNA sequence, but also detect DNA methylation. Similarly, carefully optimized mass spectrometry assays could provide proteome and metabolome data for the same single cell. To obtain high-quality integrated profiles from single cells, further improvements in the efficiencies of the assays will be essential.

### Separate

Different types of biomolecules can be biochemically extracted from the same cell lysate, separated, and independently analyzed. For example, a recent study used biotin-tagged oligo-dT adapters to pull down polyadenylated RNA, which was used for RNA-seq library preparation, while the unbound fraction was amplified and subjected to DNA sequencing [6]. This strategy critically depends on the quality of the separation because all material left in the wrong fraction is lost.

### Split

When accurate biochemical separation is not feasible, the cell lysate can be split and processed independently. For example, a recent study combined RNA and protein analysis by splitting the lysate and applying multiplex quantitative PCR for reverse-transcribed RNAs to one fraction, while affinity-based proximity extension followed by quantitative PCR for the DNA reporters of the antibodies was used for the other fraction [7]. Splitting is conceptually inferior to biochemical separation because some material will inevitably be lost in the wrong fraction, yet it is the most general strategy and feasible for many different assays.

### Convert

Biochemical conversion between different omics dimensions makes it possible to analyze them together. For example, bisulfite treatment converts DNA methylation into DNA sequence information, which can be further combined with prior treatment with a GpC methyltransferase to capture DNA methylation and nucleosome positioning in single cells [8]. It is also possible to encode information about the chromosome structure of single cells into DNA sequence information by using a protocol that ligates DNA fragments that are in close proximity in the 3D space of the nucleus [9].

### Predict

Complementary to the experimental strategies outlined above, it can also be an option to measure one or more omics dimensions directly and to predict the others using computational methods. For example, it has been shown for bulk samples that many epigenomic marks are sufficiently correlated with each other to support epigenome and transcriptome imputation, which is the inference of missing data for a given cell type from the available data for other epigenomic marks [10]. Moreover, a bioinformatic approach has been used to infer transcription factor binding and DNA copy number from single-cell DNA methylation data [11].

These five strategies provide a framework for designing ever more comprehensive multi-omics assays, given that they can be combined in many different ways. For example, a recent study used separation and conversion to link DNA methylation and gene expression profiles of the same cell [12], and DNA copy number information can also be predicted from the same data 11, 13. Further extensions may incorporate chromatin accessibility using assays such as ATAC-seq and DNase-seq, which have been optimized for single-cell profiling 14, 15. It may even be possible to split the lysed cell sample before combined DNA/RNA analysis and to subject one fraction to mass spectrometry analysis of proteins and metabolites, thus providing a first fully integrated multi-omics assay of single cells.

## Applications

Multi-omics profiling of single cells can address questions that are difficult or intractable for other methods. For example, it is becoming possible to dissect complex tissues and cellular lineage hierarchies in a data-driven manner, which complements the classical approach based on fluorescence-activated cell sorting and lineage tracing. For single-cell tissue profiling, the sample of interest is dissociated into single-cell suspension, each single cell is profiled for the relevant omics dimensions, and the profiles are bioinformatically arranged into a detailed map of the studied system (Box 1). Such data-driven dissection of cellular differentiation is both technically and bioinformatically challenging, but it holds the promise to overcome some of the problems of the classical approach, most notably its dependence on surface markers and its labor intensity.

Data-driven analysis of complex tissues and entire organs may challenge our current concept of cell types. With the resolution and throughput of single-cell assays, we may identify numerous cell states that are connected to each other through a landscape of meta-stable intermediate states, rather than a handful of stable and distinct cell types. Unfortunately, current single-cell omics assays do not retain information on cell–cell contacts and location within the tissue, but this could be addressed by combining multi-omics profiling with whole-tissue imaging or alternatively by new assays that deliver sequencing barcodes into tissue before homogenization.

Another key application of multi-omics profiling will be medicine. For many tumors, regional tumor subdivisions will vary in drug resistance, relapse, and metastasis, and comprehensive single-cell data sets may provide sufficiently detailed maps to identify the biological basis for such differences within a tumor. Assaying several omics dimensions in parallel can help uncover alternative routes to drug resistance, for example based on genetic versus epigenetic alterations, and may thereby contribute to adaptive and personalized therapy.

A third promising application of multi-omics profiling is the study of unculturable bacteria in the context of biotechnology and ecosystems research. For these bacteria, it is usually difficult to obtain a sufficiently pure population for bulk profiling, and single-cell protocols will be critical for integrative analysis, such as connecting a certain proteomic machinery to its associated metabolite profile.

Finally, the ability to measure different aspects of cell state within the same cell can be expected to uncover relevant links between the genome, epigenome, transcriptome, proteome, and metabolome of the cell. For example, it will be interesting to see how transcriptional bursts within single cells correlate with mRNA translation and protein levels, and which epigenetic changes reflect the frequency and intensity of these bursts. Similarly, single-cell multi-omic profiling may shed light on the complex relation between DNA methylation, chromatin accessibility, and transcription initiation.

## Concluding Remarks

The first single-cell multi-omics assays are now available and herald single-cell systems biology as an exciting new field of research. We predict that a renewed focus on single cells as a centerpiece of biology will create fundamental insights and interesting opportunities for practical use in biotechnology and biomedicine.

## Figures and Tables

**Figure I:**
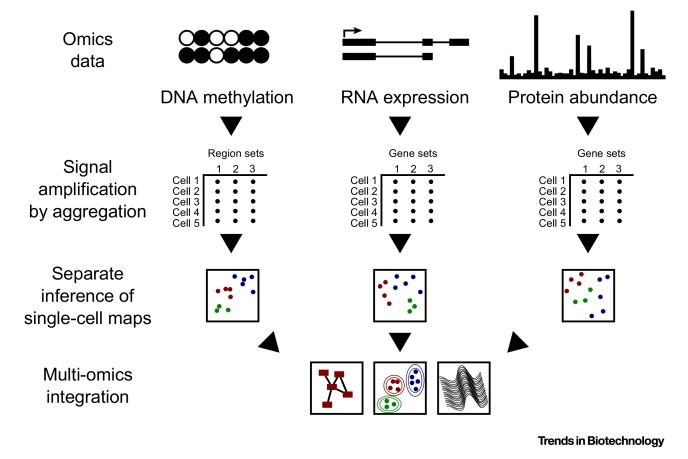
Schematic of Multi-Omics Data Analysis and Integration.

## Box 1 Bioinformatic Analysis of Multi-Omics Data in Single Cells

Multi-omics assays provide a basis for data-driven reconstruction of complex tissues and cellular lineage hierarchies using computational methods (Figure I). A generic and broadly applicable bioinformatic analysis workflow may look as follows: first, the raw data are preprocessed, filtered, and quality-controlled separately for each assayed omics dimension, accounting for the analytical challenges of single-cell data, such as technical variation, sparse signal, and amplification artifacts [2]. Second, because single-cell data are inherently low coverage, it is advisable to increase the signal:noise ratio by aggregating data; for example, by combining the expression levels of genes with similar function or DNA methylation levels across genomic regions that are bound by the same transcription factors [11]. Third, the aggregated matrix provides the input for visualizing the relative similarity and differences between single cells according to each omics dimension independently. Finally, the data are integrated into a single multi-omics map, providing a data-driven model of the studied system.
